# Haploid females in the isomorphic biphasic life-cycle of *Gracilaria chilensis* excel in survival

**DOI:** 10.1186/s12862-018-1285-z

**Published:** 2018-11-20

**Authors:** Vasco M. N. C. S. Vieira, Aschwin H. Engelen, Oscar R. Huanel, Marie-Laure Guillemin

**Affiliations:** 1 MARETEC, Instituto Superior Técnico, Universidade Técnica de Lisboa, Av. Rovisco Pais, 1049-001 Lisboa, Portugal; 2 CCMAR, Center of Marine Science, University of Algarve, Campus Gambelas, 8005-139 Faro, Portugal; 3 Instituto de Ciencias Ambientales y Evolutivas, Facultad de Ciencias, Universidad Austral de Chile, Casilla 567, Valdivia, Chile; 4 Facultad de Ciencias Biológicas, PontificiaUniversidad Católica de Chile, Santiago, Chile; 5 CNRS, Sorbonne Universités, UPMC University Paris VI, UMI 3614, Evolutionary Biology and Ecology of Algae, Station Biologique de Roscoff, CS 90074, Place G. Tessier, 296888 Roscoff, France

**Keywords:** Allee effect, Age, Competition, Density, Fertility, Life-cycle evolution, Population dynamics, Sex, Size

## Abstract

**Background:**

Conditional differentiation is one of the most fundamental drivers of biodiversity. Competitive entities (usually species) differ in environmental or ecological niche enabling them to co-exist. Conditional differentiation of haploid and diploid generations is considered to be a requirement for the evolutionary stability of isomorphic biphasic life-cycles and the cause for the natural occurrence of both phases at uneven abundances. Theoretically, stage dependent survival rates are the most efficient way to explain conditional differentiation.

**Results:**

We tested for conditional differentiation in survival rates among life stages (haploid males, haploid females, and diploids) of *Gracilaria chilensis,* an intertidal red alga occurring along the Chilean shores. Therefore, the fate of individuals was followed periodically for 3 years in five intertidal pools and, for the first time in isomorphic red algae, a composite model of the instantaneous survival rates was applied. The results showed the survival dependency on density (both competition and Allee effects), fertility, age, size, season and location, as well as the differentiation among stages for the survival dependencies of these factors. The young haploid females survived more than the young of the other stages under Allee effects during the environmentally stressful season at the more exposed locations, and under self-thinning during the active growth season. Furthermore, fertile haploid females had a higher survival than fertile haploid males or fertile diploids.

**Conclusions:**

Here, we show a survival advantage of haploids over diploids. The haploid females probably optimize their resource management targeting structural and physiological adaptations that significantly enhance survival under harsher conditions. In a companion paper we demonstrate a fertility advantage of diploids over haploids. Together, the survival and fertility differentiation support the evolution and prevalence of biphasic life-cycles.

**Electronic supplementary material:**

The online version of this article (10.1186/s12862-018-1285-z) contains supplementary material, which is available to authorized users.

## Introduction

Almost all algae (green, brown and red) have complex biphasic life-cycles, also known as haploid-diploid life-cycles, that alternate between free-living diploid (tetrasporophytes) and haploid (gametophytes) phases. On the other hand, somatic development occurs only in the diploid phase in animals and land plants (except moss and ferns). A dramatic reduction of the haploid phase is observed during the development of land plants and the multicellular haploid generation went extinct in vascular plants roughly 400 million years ago [1, 2]. The loss of the complex biphasic life-cycle in land plants has classically been related to adaptation to aerial and potentially desiccating habitats, but various evolutionary scenarios are still debated [2]. Alternatively, haploid-diploid life-cycles may have been retained in algae because they conferred functional advantages. However, the evolutionary stability of the biphasic life-cycle in algae is puzzling ecologists and evolutionists. It has been argued that niche partitioning among phases is what sustains this stability [3]. In algae characterized by morphologically highly distinct haploids and diploids (i.e. heteromorphic biphasic life-cycle), clear ecological differentiation has been observed between phases [4]. Phase differentiation is much less understood in isomorphic biphasic life-cycles, where the adult gametophytes and tetrasporophytes are virtually indistinguishable. As a consequence of isomorphicity, the sympatric haploid and diploid generations should theoretically show balanced field abundances (i.e, even ratios of haploids-to-diploids, H:D, also known as gametophyte-to-tetrasporophyte ratio, G:T). However, the commonly observed unbalance in the field has been taken as evidence of ploidy differentiation [5–10]. Different cytological processes of spore production [11, 12] and niche partitioning through conditional differentiation of their ecophysiological responses to environmental stimuli [5, 6, 8, 13–18] have been proposed to lead to unbalanced ploidy rates and abundances in haploid-diploid populations. From this conditional differentiation of phases follows that one phase should be more competitive in one set of environmental condition while the other phase should be superior under a different set; and fine-scale difference in habitat characteristics allow both gametophytes and tetrasporophytes to co-exist sympatrically. Several studies have shown ecophysiological and/or subtle morphological differences among life-cycle stages [5, 13–18] and demonstrated some differentiation between haploid and diploid vital rates [6, 7, 9, 10, 14, 16, 17]. However, in most of these studies, the performed analyses were fragmented, focusing only on specific aspects (such as spore viability, resistance to herbivory or photosynthetic performance), and several were based upon laboratory experiments rather than measures under natural conditions. Consequently, they were unable to demonstrate the occurrence in the field of the conditional differentiation among the isomorphic adults that can only be unveiled by an integrative analysis. The few remaining studies that attempted this integrative analysis [6, 7, 9, 10] found differences among life-cycle stages regarding fertility, growth or survival rates as predicted by Hughes and Otto hypothesis [3]. However, most have addressed the whole life-cycle with insufficient resolution to unveil stage differentiation within each demographic aspect.

Survival (or mortality) is one of the fundamental events in a life cycle. It is determinant for the life expectancy and has as well a strong influence on the reproductive value of an individual (i.e. its contribution to the next generation) [19]. The classical approaches for modeling demography aggregate individuals in state variables assuming homogeneity within each variable [19]. Although advantageous for their simplicity and lighter calculus, these models are unable to describe and simulate with detail the heterogeneity among individuals that leads to the complexity of the population dynamics, hence veiling holistic details. Moreover, these classical approaches are not well suited for congregating the drivers of mortality, as only a case-by-case approach can merge the factors external to an individual, but common to the population (like population density), with the internal properties of an individual (such as age, size or sex). As these internal and external factors interact in a multiplicative process, we followed the methodology applied in fisheries science [20, 21] by using instantaneous survival rates.

We monitored individuals of the red alga *Gracilaria chilensis* (Greville) Bird, McLachlan & Oliveira, periodically over 3 years. Each individual was tagged and its properties followed from birth to death. This data set represents an excellent opportunity to infer if and how diploids and haploid males and females differ in fundamental vital rates: fertility, growth, and survival. Here, we tested for conditional differentiation of survival rates among life-cycle stages of *G. chilensis*. The several drivers of differentiated survival were accessed applying a composite model of the instantaneous survival rates.

## Methods

### Demographic data

**Fig. 1:**
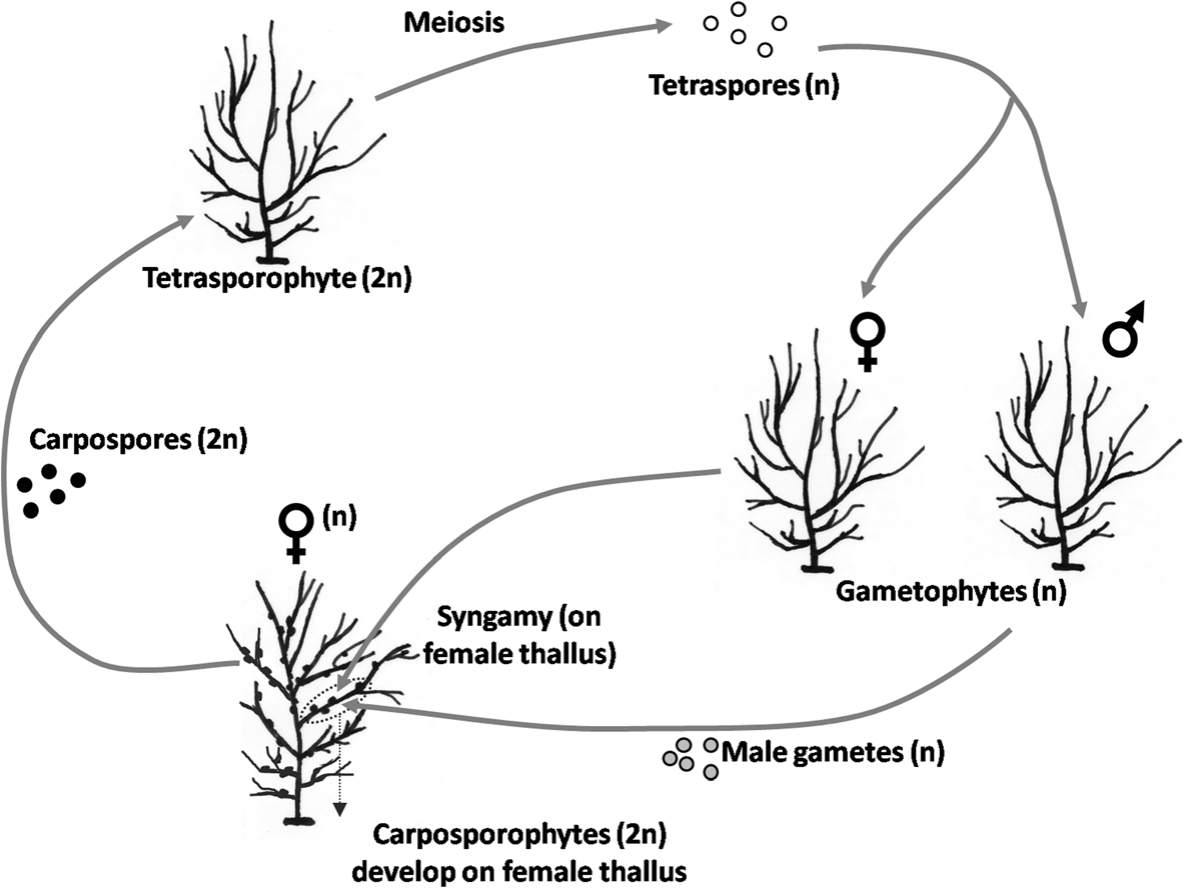
Life-cycle. The typical haploid-diploid life cycle of *Gracilaria* species, with the alternation of meiosis and syngamy connecting tetrasporophytes and gametophytes individuals (modified from Kain & Destombe [61] and Guillemin et al. [62]

*Gracilaria chilensis* is a red macroalga occurring in the intertidal along the Chilean shore that plays a highly relevant role in the agar market worldwide. Individuals are fixed to rocky bottom by a holdfast and may survive and re-grow new fronds after the older were lost [22]. This species has a complex isomorphic biphasic life-cycle (Fig. 1), also known as haploid-diploid life-cycle, alternating free living tetrasporophytes (diploid) and gametophytes (haploid). The gametophyte males release gametes that fertilize the gametophyte females. From the fertilized oogonia develops a short-lived diploid epiphytic stage (the carposporophyte), acquiring nutrients from the female gametophyte for the development and production of diploid spores (i.e., carpospores) [23]. The carpospores are released into the environment where they settle and develop to become tetrasporophyte adults, which in their turn produce haploid tetraspores after meiosis and release them to the environment. Settled tetraspores grow to become adult male or female gametophytes, thus closing the cycle.

Demographic monitoring of individuals was performed in 5 intertidal rock-pools (‘Corral 1’, ‘Corral 2’, ‘Niebla 1’, ‘Niebla 2’ and ‘Niebla 3’) within 2 sites (Corral 39°52′27″S / 73°24′02″W and Niebla 39°55′47″S / 73°23′57″W) along the margins of the Valdivia river estuary. These pools corresponded to *G. chilensis* stands in the upper intertidal. The Niebla stands were in rock-pools that preserved some amount of water during low tide. The Corral stands where on rocky platforms presenting a gentle slope and the individuals dried on the bare rock during low tide. Sampling was performed from October 2009 to February 2011 at 4-month intervals. The interval between February and June mostly comprehends the austral autumn, the interval between June and October mostly comprehends the austral winter and the interval between October and February comprehends the austral spring and summer. All individuals within each rock-pool were mapped to a pair of fixed points. A small piece of tissue was collected from each individual. Males (M), females (F) and tetrasporophytes (D for diploids) were identified using first the observation of reproductive structures under a binocular microscope, and the sex-specific molecular markers for the remaining vegetative individuals [24]. Frond length and diameter were recorded for each individual observed at each census. The volume (*v*_*i*_) of a cylinder of equal length and diameter was used as a proxy for ramet biomass measured as dry weight (*r*^*2*^ = 0.877; *p* < 0.0001; *n* = 281). Every individual absent after 4 months was re-checked after 8 months for confirmation and considered as dead when missing in the re-check. The sampling sites were in public land, no specific permissions were required for these locations and activities, *G. chilensis* is not an endangered or protected species, and the sampling method was non-destructive.

### Composite survival rates

**Fig. 2:**
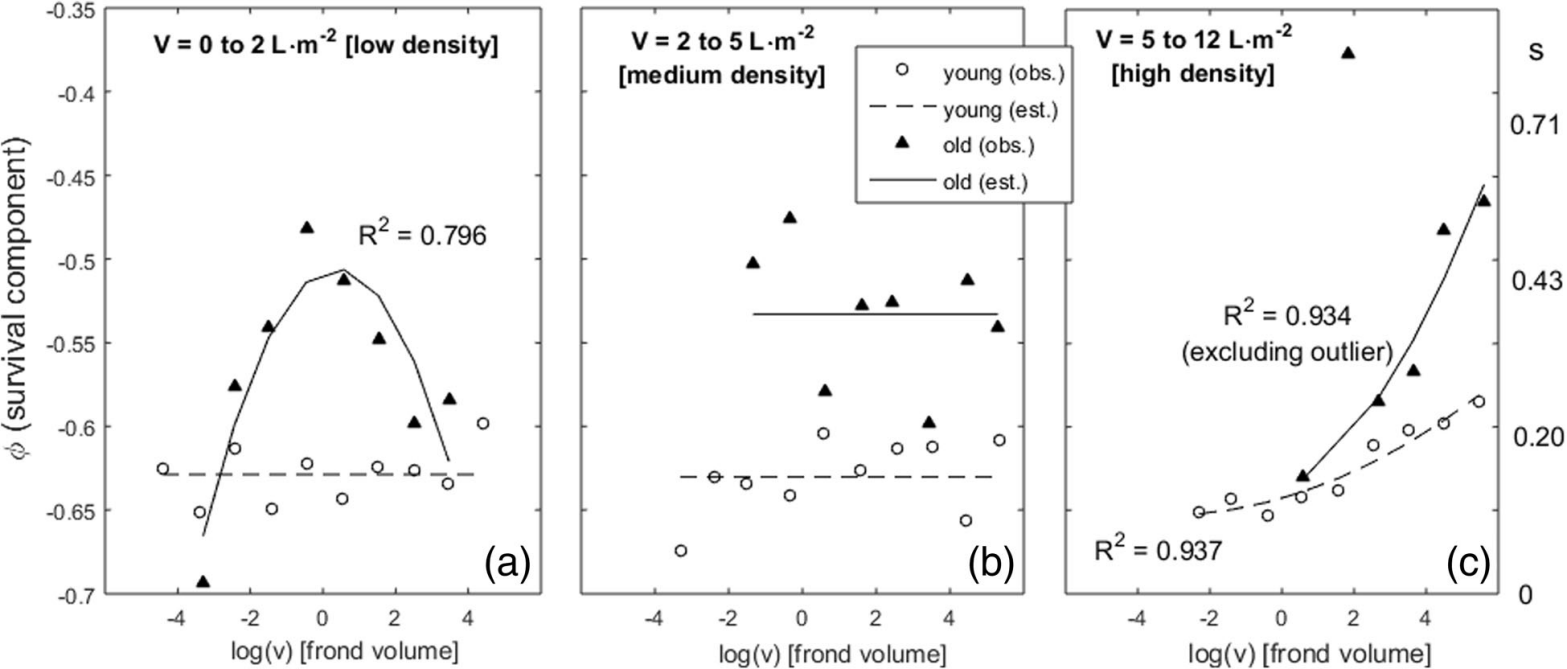
General dynamics of the age, size and density-dependent survival (φ) of *G. chilensis* individuals with fronds. Each panel (**a**, **b** and **c**) corresponds to a range of algal stand density (V). The φ is shown in the left y-axis and its corresponding finite survival rate (s) in the right y- axis. Individuals grouped according to their ages as < 8 month (young) or > 8 month (old). Frond volume (v) given in cm^3^. The line-fits with poor non-linear correlation coefficients (*R*^*2*^ < 0.5) were replaced by their group mean

The finite survival rate (*s*_*i*_) of ramet *i* in pool *p* was transformed into *ṡ* by applying the logit function. This was required because the observations of *s* = 0 disabled the estimation of the composite survival model parameters, as explained in the paragraph below. The *ṡ* had a corresponding instantaneous survival rate given by *ṡ*_*i*_ = *e*^*yi*^. This was decomposed into its several forcings by *y*_*i*_ = φ_*i*_ + η_*i*_, where η_*i*_ accounted for the cost of fertility on the survival of an individual and φ_*i*_ accounted for the effects of age, size and algal stand density. This model could be applied differently to each stage, pool and season (Fig. 2). Following the standard in fisheries science [20, 21], the factors driving survival were assumed to interact in a multiplicative way applied over finite rates and thus additive when applied over their derived instantaneous rates i.e., *e*^φ*i +* η*i*^ = *e*^φ*i*^*∙e*^η*i*^ = *ṡ*_i_. First, the effects on survival of age, size and location were estimated from the instantaneous survival rate of each individual (*y*_*i*_) assuming that η_*i*_ = 0. Next, the effect of fertility (η_*i*_) was estimated by η_*i*_ = *y*_*i*_-φ_*i*_.

The parameter estimation could not use observations of *s* = 0, in which case the resulting *y* = −∞ would disable posterior calculations. Since we worked with individual survival, which could only be 0 or 1, *s* had to be transformed into *ṡ* by applying the logit function, *ṡ* = exp.(*s*)/(1 + exp.(*s*)); with the observations of individual survival becoming *ṡ* = 0.5 or *ṡ* = 0.73. The estimation of the composite survival was applied to *ṡ* and, afterwards, survival was reverse calculated as *s* = log(*ṡ*/(1-*ṡ*)). At each step of the iterative estimation of φ_*i*_ and η_*i*_ we ascertained that log(0.5) < *y* < log(0.73), and thus 0 < *s* < 1. Failure of this quality test was indicative that the current model estimation was not taking into account fundamental interactions among the drivers of mortality/survival.

Some individuals temporarily consisted of bare holdfasts, either because they were germlings or because their fronds had detached and new ones had not grown yet. *G. chilensis* holdfasts are small, prostrated encrusting disks extremely difficult to detect in the field when fronds are not present. Hence, the sampling procedure only detected these individuals when they survived and grew new fronds. Because we only detected the bare holdfasts that survived, their observed survival rate always equaled 1. To prevent this bias, the survival dynamics of holdfasts without fronds was excluded from the remaining analysis.

#### Age, size and density dependent survival φ

The age of an algal ramet is identical to the age of its holdfast, which corresponds to the perennial part of the individual. Due to the temporal turnover of fronds (i.e. that can be considered as ephemeral) the age of a ramet is not necessarily the age of its current frond. The overwhelming majority of the individuals with fronds corresponded to younger ramets with small fronds. Hence, an analysis unbiased towards the young required grouping individuals according to age×size classes and estimating the φ_i_ using their group mean. Then, all classes accepted were equally weighted, irrespective of the number of observations within each of them. Age was aggregated by age ≤ 2 projection intervals (i.e., ≤8 month, reported as “young”) and age > 2 projection intervals (i.e.,> 8 month, reported as “old”). The frond sizes were given by their volumes (*v*) in cm^3^, with the size aggregation given by the − 6 < log(*v*) < 6 at intervals of 1 or sometimes 2. The φ of each of the two age classes was fit to a second-degree polynomial dependent from log(v) i.e., φ = a + b∙log(v) + c∙log(v)^2^. These relations were also dependent on the stand density, and thus they were tested for three stand densities aggregated within V = [0 2], V = [2;5], and V= [5, 12] L∙m^2^. V is the sum of all frond volumes (v) in the pool, excluding the larger frond to prevent bias from the random occurrence of exceptionally large individuals, and standardized by pool area. The fundamental role of density in the demography of *G. chilensis* has previously been demonstrated [25].

#### Fertility-dependent survival η_*i*_

Above a certain age and size threshold, almost all individuals were fertile, whereas the infertile tended to be younger and smaller. Hence, it was not feasible to compare fertile to infertile individuals in an ANOVA design. Fertility-dependent survival was tested separately for fertile and infertile individuals using permutation tests with 3-way orthogonal ANOVA designs (stage×pool×season) and 10,000 permutations. Factors ‘stage’ and ‘season’ were fixed effects and factor ‘pool’ was random effects. Third order interactions could not be determined as they lacked replication within. The fertility-dependent survival were estimated from the respective fertile and infertile ANOVA linear models setting α = 0 for the factors and/or interactions that were found non-significant.

## Results

The survival dependency from age, size and stand density (φ) showed a dynamics with several conspicuous features matching well-established demographic characteristics of plants and seaweeds (Fig. 2):

i) older individuals had higher survival probabilities than younger individuals, with the latter always showing low survival probabilities.
ii) very low densities were detrimental to survival; a feature known as Allee effects. Under these circumstances, the survival of older individuals was optimized at intermediate sizes, whereas both size extremes were detrimental (Fig. 2a).
iii) under high densities, survival was proportional to frond size (Fig. 2c). The survival of the smaller decreased whereas the survival of the larger increased when compared to the size-even survival observed under moderate densities (i.e., compared to Fig. 2b). This is the expected from the self-thinning law, which states that under active growth the stands get crowded subjecting individuals to intense competition; with the larger and stronger overcoming the smaller and weaker.

**Fig. 3:**
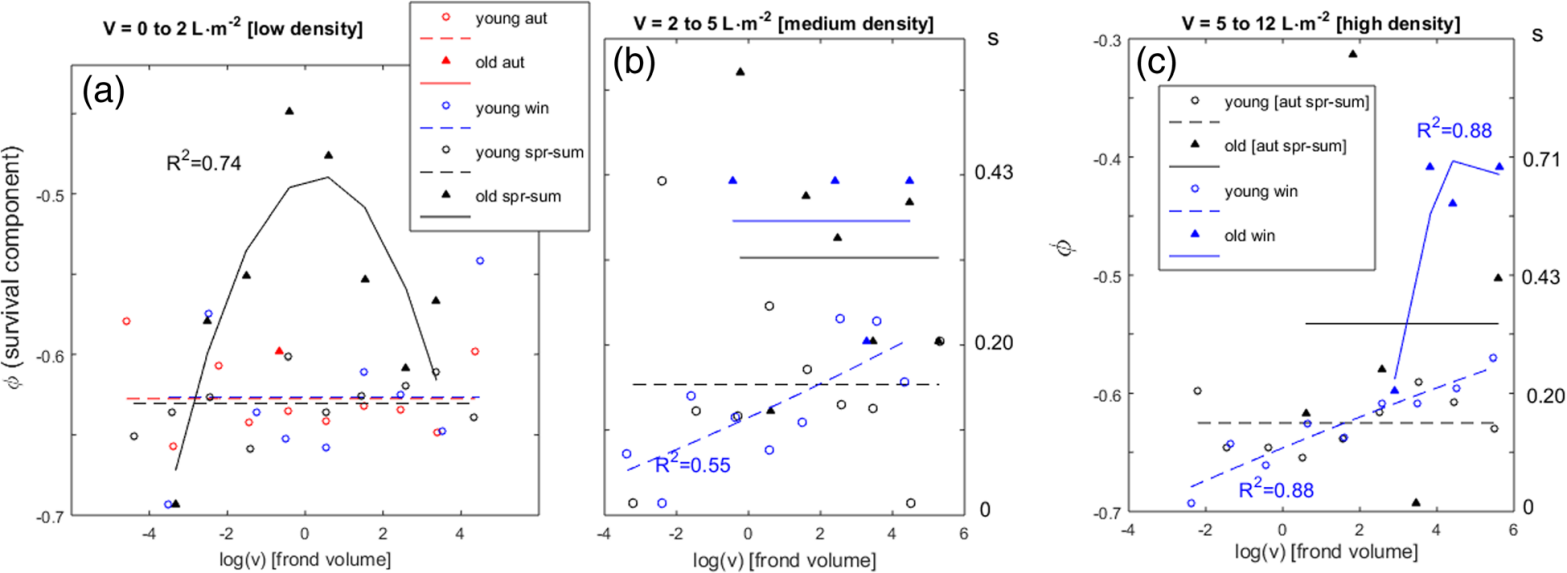
Seasonal dynamics of the age, size and density-dependent survival (φ) of *G. chilensis* individuals with fronds. Seasons are spring-summer (spr-sum), autumn (aut) and winter (win). Each panel (**a**, **b** and **c**) corresponds to a range of algal stand density (V). The φ is shown in the left y-axis and its corresponding finite survival rate (s) in the right y-axis. Individuals were grouped according to their ages as < 8 month (young) or > 8 month (old). Frond volume (v) given in cm^3^. The line-fits with poor non-linear correlation coefficients (*R*^*2*^ < 0.5) were replaced by their group mean. Only groups with sufficient data for reliable estimates are shown. Panels (**a**, **b**) share the same scales. Panels (**b**, **c**) share the same legend

The survival dynamics, with density- and age-dependent components that revealed Allee effects and self-thinning, was then tested for seasonality (Fig. 3). *G. chilensis* shows active growth, with stands getting crowded during the winter season. Hence, self-thinning took place during the winter season while affecting individuals of all ages (Fig. 3c). Furthermore, during this season the younger individuals were affected by self-thinning even when living under moderate densities (Fig. 3b). During the spring-summer season, environmental stress replaced competition as the fundamental cause of mortality. Indeed, survival during spring-summer season was dependent from stand density, with the occurrence of Allee effects (Fig. 3). Younger individuals consistently occurred under very low population densities during any season (Fig. 3a) and always shown very low survival rates.

**Fig. 4:**
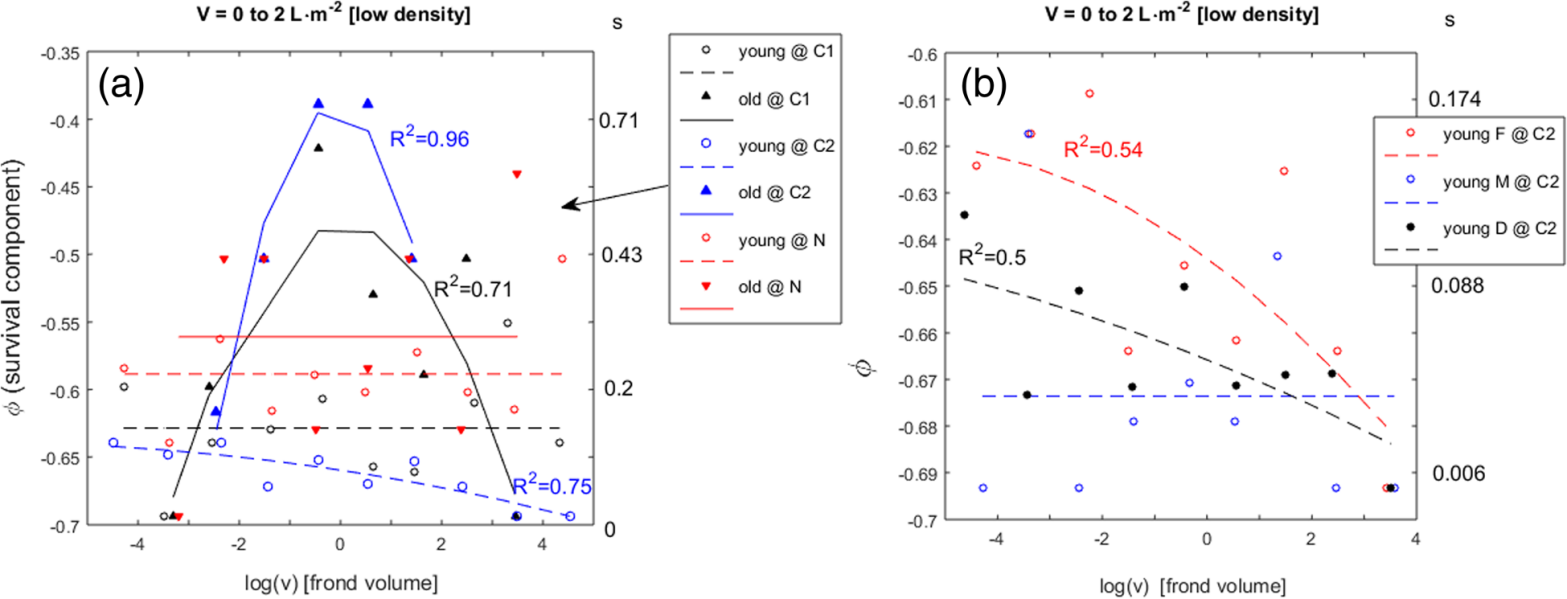
Allee effects over thesurvival (φ) of *G. chilensis* individuals with fronds. Each panel (**a**, **b** and **c**) corresponds to a range of algal stand density (V). The φ is shown in the left y-axis and its corresponding finite survival rate (s) in the right y-axis. Individuals were grouped according to their ages as < 8 month (young) or > 8 month (old). Frond volume (v) given in cm^3^, according to their stage as males (M), females (F) or diploids (D), and according to their pool as Corral 1 (C1), Corral 2 (C2), Niebla 1(N1), Niebla 2 (N2) and Niebla 3 (N3). The line-fits with poor non-linear correlation coefficients (*R*^*2*^ < 0.5) were replaced by their group mean

The Allee effects, occurring only during the environmentally harsher spring-summer season, were then analyzed regarding the effects of location and ploidy (Fig. 4). Under similar population densities, the strength of the Allee effects varied with location, being the strongest at Corral 2 (C2), a little weaker at Corral 1 (C1), and the weakest at the Niebla (N) pools (Fig. 4a). Stronger Allee effects increased the differentiation in survival between young and old as well as the sensitivity of both size extremes (Fig. 4a at the Corral 2 pool). On the other hand, the absence of Allee effects drove individuals of different ages into evening their survival rates (Fig. 4a, at the Niebla pools). Under the strongest Allee effects (at the Corral 2 pool) a differentiation among stages was visible: the survival of the young and small haploid females prevailed over the survival of the young and small diploids, which in their turn was higher than the survival of the young and small haploid males (Fig. 4b).

**Fig. 5:**
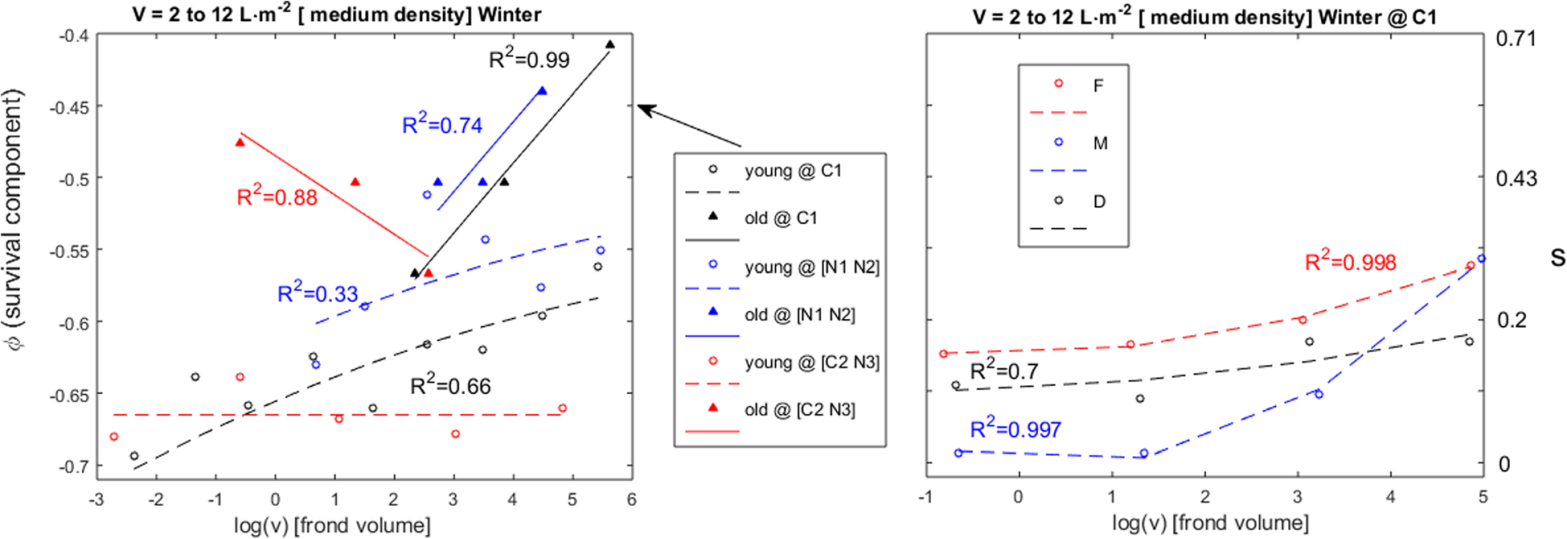
Self-thinning effects over the survival of *G. chilensis* individuals with fronds. Each panel corresponds to a range of algal stand densities (V). The φ is shown in the left y-axis and its corresponding finite survival rate (s) in the right y-axis. Individuals grouped according to their ages as < 8 month (young) or > 8 month (old). Frond volume (v) given in cm^3^, according to their stage as males (M), females (F) or diploids (D), and according to their pool as Corral 1 (C1), Corral 2 (C2), Niebla 1(N1), Niebla 2 (N2) and Niebla 3 (N3)

The self-thinning general dynamics, occurring only during the winter growth season and with an age-dependent effect, was analyzed for effects of location and ploidy (Fig. 5). Self-thinning was only observed in the Corral 1, Niebla 1 and 2 pools. In the Corral 2 and Niebla 3 pools, the young were observed at high densities and always showed very low survival, whereas the older individuals were only observed at moderate densities. The number of older individuals within the Corral 1, and Niebla 1 and 2 pools was too small to split them into stage classes and perform robust testing. In the Corral 1 pool, stages showed differences while self-thinning, with enhanced survival of the haploid females. In order to obtain the best linefits specific to each stage the data from moderate and high densities were merged.

In most of the situations where Allee effects or self-thinning were absent it was still possible to observe differences in the survival rates among pools and seasons. Yet, these results were subsidiary and we chose not to present them. The age-, size- and density-dependent survival (φ) relations derived above were congregated in a general algorithm used to estimate the φ_i_- i.e., specific to each individual at each census - and subsequently the η_i_ = ṡ_i_-φ_i_.

The fertility-dependent survival components η exhibited means of μ_infer_ = − 0.0011 and μ_fer_ = 0.001 for the infertile and fertile fronds, respectively. Their difference ∆η = 0.0021 was much smaller than their error variances σ^2^_infer_ = 0.019 and σ^2^_fer_ = 0.021, and a permutation test confirmed that this difference was not significant (*p* = 0.746, df_factor_ = 1, df_error_ = 2762). Despite their resemblance in mean values, their internal data structures - i.e., the fertility-dependent survival (η) dispersal pattern and correlation with its forcing functions - were quite different. Indeed, separate permutation tests (Table 1) revealed that the η of the fertile fronds showed a conspicuous differentiation among life-stages that the η of the infertile fronds did not show. The fertile haploid females survived more (η_F_ = 0.0127) than the fertile haploid males (η_M_ = − 0.0117, p_F ≠ M_ = 0.004) or the fertile diploids (η_D_ = − 0.0013, p_F ≠ D_ = 0.064). Estimating the composite survival model for the fertile fronds, with these η and the average φ, yield survival rates of s_F_ = 0.19, s_M_ = 0.13 and s_D_ = 0.15. The fertile females survived 40% better than the fertile males and 20% better than the fertile diploids.

**Table 1:** Effects on the fertility-dependent (η) survival

Factors	η infertile			η fertile		
	d.f. error	d.f. factor	*p*	d.f. error	d.f. factor	*p*
Stage	745	2	0.9859	2015	2	**0.0135**
Pool	743	4	0.1662	2013	4	0.5129
Season	745	2	0.2345	2015	2	0.3828
Stage×pool	739	8	0.7576	2009	8	0.8099
Stage×season	743	4	0.3358	2013	4	0.1254
Pool×season	739	8	0.5061	2009	8	0.4960

Permutation tests for the significances of the effects on the fertility-dependent (η) survival components estimated separately for fertile and infertile fronds; 10.000 iterations for each test (i.e, the original + 9999 randomizations) were used. Significant *p*-value in bold

**Fig. 6:**
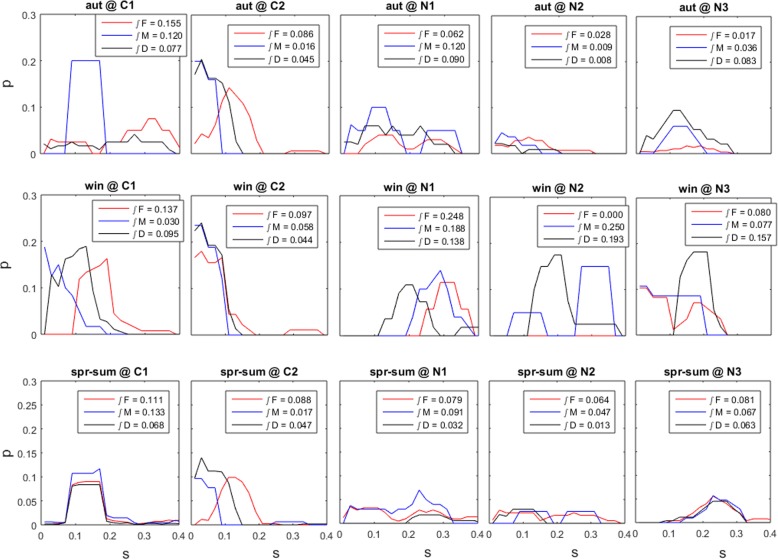
Survival probability density functions. Survival (s) estimated by the composite survival model for the life cycle stages males (M), females (F) and diploids (D) at each pool and during each season. Integrals (∫) correspond to average probabilities of survival i.e. ∫p∙s. Pools are Corral1 (C1), Corral2 (C2), Niebla1 (N1), Niebla2 (N2) and Niebla3 (N3). Survival observed during the spring-summer (spr-sum), autumn (aut) and winter (win)

Using the η_i_ and φ_i_, we estimated the instantaneous survival rate for each individual at each census interval. Then, the logit function was applied backwards to retrieve the predicted finite survival rate. Clustering individuals by stage, pool and season, and estimating the respective survival probability distributions enabled a clear perspective of the haploid dominance over diploids in this aspect of the life-cycle (Fig. 6). The observed generalized survival of *G. chilensis* was low. Haploids tended to have a higher survival than diploids, particularly when the survival rates were amongst the lowest observed. Diploid individuals were outperformed by the haploid females in 73% and by the haploid males in 67% of the pool×season combinations.

## Discussion

Conditional differentiation is considered as a requirement for the stability and evolution of isomorphic biphasic life-cycles [3] and, except when haploids are favoured in some situations and diploids in other, haploid-diploid life cycles will rapidly evolve toward diplonty or haplonty. Theoretically, this conditional differentiation should be most efficient when acting over survival rates [7, 26–29]. However, only part of the studies made on haploid-diploid algae has been able to detect difference in survival rates among life-cycle stages in the field [not detected: 6,7; detected: 9,30]. For example, even in studies sustained by extensive field monitoring [6, 7], the estimation of the overall survival rates has not allowed the detection of any clear survival differences between stages in *Gracilaria* populations from the Atlantic. On the other hand, our study reveals clear differences in survival among different life-history stages in natural populations of *G. chilensis*, thereby supporting the role of conditional differentiation in the maintenance of the haploid-diploid life-cycle of this species. Here, we showed that haploid females survive better than any other life history stage, especially when resource management becomes critical for survival. The young haploid females survived better than the young haploid males or the young diploids under stress from competition during the active growth season, and under stress from Allee effects during the environmentally stressful spring-summer season. Supporting our results, a haploid superiority regarding the survival of *G. chilensis* had previously been observed under laboratory experiments for juvenile fronds subject to light and salinity gradients [22]. Furthermore, our results also showed that the haploid females endure better the fertility-related mortality than the haploid males or the diploids. Regardless of the tide pool, season or maturity state, a survival advantage of diploids was never observed, implying that this ploidy stage must present an advantage in some other aspect of the life-cycle. *G. chilensis* diploids have been observed to completely dominate populations maintained only or mainly by asexual reproduction (i.e. budding) and vegetative growth [31]. Moreover, in the companion article (Vieira VMNCS, Engelen AH, Huanel O, Guillemin M-L: Differentiation of haploid and diploid fertilities in Gracilaria chilensis affect ploidy ratio, submitted) we demonstrate a fertility advantage of diploids over haploids. Differences between phases for distinct vital rates with haploids better at survival and diploids showing higher growth and fertility support the importance of conditional differentiation on evolution and maintenance of biphasic life-cycles.

During the environmentally stressful spring-summer season, both the very small and the very large fronds suffered intense Allee effects - i.e., when population densities are so low that they negatively affect individual survival rates and consequently the population dynamics. We hypothesize the increased mortality of the smaller individuals is due to desiccation and UV, and the increased mortality of the larger individuals to be due to hydrodynamic stress from current drag. However, significant differences in survival between stages were only observed for the smaller fronds and benefiting the young haploid females, while larger fronds of all stages exhibited equally poor survival rates. Allee effects have previously been observed in haploid-diploid intertidal algae and have been associated with highly negative impacts of desiccation [8, 32–35] or hydrodynamic stress [8, 36]. Under these stressful conditions, a haploid dominance has frequently been observed in intertidal algal stands [6, 7, 30, 35, 37–40]. Interestingly, the splatter of waves in areas of high hydrodynamic stress limits the negative impact of desiccation in the intertidal [9] and some evidence support that the haploid advantage could be more related to resistance to desiccation than wave action [8, 35, 41, 42]. For example, in the case of *Mazzaella oregona,* which occurs in the intertidal at low population densities, diploids dominate in wave-exposed sites whereas haploids dominate in wave-protected sites (i.e. where the absence of splash turns them prone to desiccation) [8]. Similarly, in intertidal populations of *M. splendens* located in wave-protected sites, diploids dominate during winter whereas haploids dominate during summer [42]. This seasonal pattern has been associated to changes in air temperature and desiccation. For intertidal *G. chilensis*, intense exposure to UV radiation was demonstrated as an important source of stress [18, 43]. Accordingly, Allee effects affected our intertidal pools during the spring-summer season. These pools were located at the mouth of an estuary, where wave action is limited, and corresponded to the highest intertidal *G. chilensis* parches in both Niebla and Corral. Consequently, individuals could be subject to hydrodynamic stress from currents and tides, and to intense summer desiccation. Mortality was higher in Corral, where the species live on rocky platforms presenting a gentle slope and the individuals are drying on the bare rock during low tide than in Niebla where the species was sampled from rock pools. For *G. chilensis* studied in exactly the same location 1 year after our experiment, resistance to stress from temperature and UV radiation, conferred by the accumulation of phenolic compounds, was determinant for physiological rates [18]. Fertile females produced more phenolic compounds and were less photo-inhibited than diploids when subject to UV stress and temperatures ≥10 °C during 48 h incubations. Together, our study and other studies on *G. chilensis* [18, 43, 44] evidenced that, within the intertidal, the young haploid females could have an ecological advantage over the young haploid males and young diploids due to an increased resistance to desiccation and UV. The presence of chemical compounds conferring resistance to desiccation and UV is generalized to the *Gracilaria* genus [44–48], with ecological advantage for the intertidal individuals having them in larger concentrations [47, 48].

Our results show that the young haploid females also survive better than the young diploids under crowded conditions during active growth season. In these conditions, representing an extreme opposite from Allee effects, intense intraspecific competition for space and resources occurs among thalli that lead to self-thinning, a feature well documented to affect both seaweeds and terrestrial plants ([49, 50]; Creed JC, Norton TA, Caetano D, Vieira VMNCS: A meta-analysis shows that seaweeds surpass plants setting life-on-Earth’s limit for biomass concentration, submitted). Self-thinning has generally been reported to occur seasonally; mostly impacting populations during the growing season (see review by Scrosati [51]). In *G. chilensis*, it was observed only during the austral winter. Supporting our findings, increased gametophyte dominance has been observed associated with increased population densities in other haploid-diploid algae [8, 40–42].

Finally, our results reveal that in infertile individuals no difference in survival exists between sex or ploidy states. However, when it comes to the fertile individuals, the haploid females survived better than any other fertile fronds. Differences in survival maybe related to distinct capacity to resist feeding pressure among life-cycle stages and linked to the accumulation of herbivore deterring chemical compounds in the reproductive structures of mature thalli. *G. chilensis* and other species of *Gracilaria* possess such chemical defenses [52, 53]. Furthermore, differences between life-cycle stages in these defense mechanisms have been reported and demonstrated responsible for a stage selected herbivore impact [5, 16, 17, 35, 54]. Indeed, herbivores preference for the red alga *Asparagopsis armata* goes from male gametophytes to sporophytes and at last female gametophytes [17]. Their least preferred tissue to feed on is the cystocarp growing on female thallus and Vergés et al. [17] reported that, indeed, the highest content of secondary metabolites deterring herbivores was found within the cystocarp cell wall.

Regarding the evolutionary benefits of biphasic life-cycles, other hypotheses have been advanced that are not conflictive with the hypothesis proposed by Hughes and Otto [3] but may rather constitute different aspects of a whole dynamics. Lewis [55] proposed that, when resources are scarce, haploids gain advantage from spending fewer resources producing/replicating half the DNA of diploids. This non-genetic explanation for the evolution of life-cycles, which became known as the nutrient limitation hypothesis [56], gained experimental sustain when haploids of *Gracilaria verrucosa* grew faster than diploids cultivated in nutrient-poor conditions [57]. Since then, new evidence emerged generalizing the cost of nucleotide polymer production in green, red and brown macroalgae by proving that intracellular RNA concentration is strongly dependent on nutrient availability [58]. This nutrient limitation hypothesis may explain why the *G. chilensis* haploid females excel in survival when the efficiency of resource management becomes critical, for example when competing under self-thinning or in situations where the production of costly chemical compounds that protect from desiccation, UV radiation or herbivory are key. However, in *G. chilensis* the survival of the two haploid gametophytes was highly distinct with a very low survival of males, lower even than that of the diploids, which should undermine the application of the nutrient limitation hypothesis to *G. chilensis*. Nonetheless, our concomitant work on *G. chilensis* fertility (Vieira VMNCS, Engelen AH, Huanel O, Guillemin M-L: Differentiation of haploid and diploid fertilities in Gracilaria chilensis affect ploidy ratio, submitted) showed that the stage differentiation in fecundity is completely symmetrical from the one observed in the current work on survival. It is possible, then, that haploid males alternatively allocate on fecundity their resources spared from DNA duplication. Trade-offs between fertility and survival were also detected in two other red algae from Chile [59] and in several iteroparous plants [60]. Furthermore, in all those plant species the trade-off between fertility and survival was very sensitive to the nutrient availability [60]. Differential allocation of resources to fecundity or survival depending on sex is a commonly encountered trait, especially in the animal kingdom where males produce billions of sperm while females survive longer. When females carry the earlier developmental stages of the next generation - in the case of the red algae, the cystocarps –their survival is a key element in the species’ life-cycle dynamics [19]. The survival of the males in this case can be of lesser importance, since only a few of them surviving long enough to reproduce may be sufficient to fertilize most females in the population.

## Conclusions

The survival of *Gracilaria chilensis* depends on density (both due to competition and to Allee effects), fertility, age, size, season and location, with the life-cycle stages differentiating among themselves for the survival dependencies of these factors. This finding supports the hypothesis on the necessity of conditional differentiation for the prevalence of biphasic life-cycles. The young haploid females survived more than the young of other stages under Allee effects during the environmentally stressful season at the more exposed locations, and under self-thinning during the active growth season. Furthermore, fertile haploid females had a higher survival than fertile haploid males or fertile diploids. This differentiation probably arises from the ability of the females to optimize their resource management targeting structural and physiological adaptations that significantly enhance survival under harsher conditions.

## Additional file

Additional file 1: *Gracilaria chilensis* raw data. Data comprising the life cycle stage, size and sexual maturity of each *Gracilaria chilensis* frond at each pool and each census. (ZIP 830 kb)

## Abbreviations

ANOVA: Analysis of Variance
C: Corral sampling site
C1: Corral pool #1
C2: Corral pool #2
D: Diploids
DNA: Deoxyribonucleic acid
F: Female haploids
G: Gametophytes
G:T: Gametophyte-to-tetrasporophyte ratio
H: Haploid
H:D: Haploid-to-diploid ratio
M: Male haplois
N: Niebla sampling site
N1: Niebla pool #1
N2: Niebla pool #2
N3: Niebla pool #3
RNA: Deoxyribonucleic acid
T: Tetresporophytes
UV: Ultra-violet radiation

## References

[1] Kenrick P, Crane PR (1997). The origin and early evolution of plants on land. Nature.

[2] Niklas KJ, Kutschera U (2010). The evolution of the land plant life cycle. New Phytol.

[3] Hughes JS, Otto SP (1999). Ecology and the evolution of biphasic life-cycles. Am Nat.

[4] Thornber CS (2006). Functional properties of the isomorphic biphasic algal life-cycle. Integr Comp Biol.

[5] Hannach G, Santelices B (1985). Ecological differences between the isomorphic reproductive phases of two species of *Iridaea* (Rhodophyta: Gigartinales). Mar Ecol Prog Ser.

[6] Destombe C, Valero M, Vernet P, Couvet D (1989). What controls the haploid-diploid ratio in the red alga, *Gracilaria verrucosa*?. J Evol Biol.

[7] Engel C, Aberg P, Gaggiotti OE, Destombe C, Valero M (2001). Population dynamics and stage structure in a haploid-diploid red seaweed, *Gracilaria gracilis*. J Ecol.

[8] Mudge B, Scrosati R (2003). Effects of wave exposure on the proportion of gametophytes and tetrasporophytes of *Mazzaella oregona* (Rhodophyta: Gigartinales) from Pacific Canada. J Mar Biol Assoc UK.

[9] Thornber CS, Gaines SD (2003). Spatial and temporal variation of haploids and diploids in populations of four congeners of the marine alga *Mazzaella*. Mar Ecol Prog Ser.

[10] Thornber CS, Gaines SD (2004). Population demographics in species with biphasic life cycles. Ecology.

[11] Scrosati R, DeWreede RE (1999). Demographic models to simulate the stable ratio between ecologically similar gametophytes and tetrasporophytes in populations of the *Gigartinaceae* (Rhodophyta). Phycol Res.

[12] Fierst J, terHorst C, Kubler JE, Dudgeon S (2005). Fertilization success can drive patterns of phase dominance in complex life histories. J Phycol.

[13] Gonzalez J, Meneses I (1996). Differences in the early stages of development of gametophytes and tetrasporophytes of *Chondracanthus chamissoi* (C.Ag.) Kützing from Puerto Aldea, northern Chile. Aquaculture.

[14] Garza-Sanchez F, Zertuche-Gonzalez JA, Chapman DJ (2000). Effect of temperature and irradiance on the release, attachment, and survival of spores of *Gracilaria pacifica* Abbot (Rhodophyta). Bot Mar.

[15] Carmona R, Santos R (2006). Is there an ecophysiological explanation for the gametophyte-tetrasporophyte ratio in *Gelidium sesquipedale* (Rhodophyta)?. J Phycol.

[16] Thornber C, Stachowicz JJ, Gaines S (2006). Tissue type matters: selective herbivory on different life history stages of an isomorphic alga. Ecology.

[17] Vergés A, Paul NA, Steinberg PD (2008). Sex and life-history stage alter herbivore responses to a chemically defended red alga. Ecology.

[18] Cruces E, Flores-Molina MR, Díaz MJ, Huovinen P, Gómez I (2018). Phenolics as photoprotective mechanism against combined action of UV radiation and temperature in the red alga *Gracilaria chilensis*?. J Appl Phycol.

[19] Caswell H. Matrix population models: construction, analysis and interpretation. Sunderland: Sinauer Associates; 2001.

[20] Allen MS, Miranda LE, Brock RE (1998). Implications of compensatory and additive mortality to the management of selected sportfish populations. Lakes Reserv Res Manag.

[21] Allen MS, Walter CJ, Myers R (2008). Temporal trends in largemouth bass mortality, with fishery implications. North Am J Fish Manage.

[22] Guillemin Marie-Laure, Sepúlveda Roger D., Correa Juan A., Destombe Christophe (2012). Differential ecological responses to environmental stress in the life history phases of the isomorphic red alga Gracilaria chilensis (Rhodophyta). Journal of Applied Phycology.

[23] Kamiya M, Kawai H (2002). Dependence of the carposporophyte on the maternal gametophyte in three ceramiacean algae (Rhodophyta), with respect to carpospore development, spore production, and germination success. Phycologia.

[24] Guillemin M-L, Huanel OR, Martínez EA (2012). Characterization of genetic markers linked to sex determination in the haploid-diploid red alga Gracilaria chilensis. J Phycol.

[25] Vieira V, Engelen AH, Huanel OR, Guillemin M-L (2016). Linear-in-the-parameters oblique least squares: a case study with the estimation of density-dependent survival in algae with isomorphic biphasic life-cycles. PLoS One.

[26] Vieira VMNCS, Santos ROP (2010). Demographic mechanisms determining the dynamics of the relative abundance of phases in biphasic life cycles. J Phycol.

[27] Vieira VMNCS, Santos ROP (2012). Responses of the haploid to diploid ratio of isomorphic biphasic life cycles to time instability. J Biol Dyn.

[28] Vieira VMNCS, Santos ROP (2012). Factors that drive the geographical variability of the haploid:diploid ratio of biphasic life cycles. J Phycol.

[29] Vieira VMNCS, Mateus MD (2014). Regulation of the demographic structure in isomorphic biphasic life cycles at the spatial fine scale. PLoS One.

[30] Bhattacharya D (1985). The demography of fronds of *Chondrus crispus* Stackhouse. J Exp Mar Biol Ecol.

[31] Faugeron S, Destombe C, Viard F, Correa JA, Valero M (2008). Domestication and distribution of genetic variation in wild and cultivated populations of the haploid diploid red alga gracilaria chilensis: how a traditional framing practice favour asexual reproduction and heterozygocity?. Evolution.

[32] Olson AM (1990). Algal life history stages respond differently to desiccation and herbivory (abstract). Bull Ecol Soc Am.

[33] Scrosati R, DeWreede RE (1998). The impact of frond crowding on frond bleaching in the clonal intertidal alga *Mazzaella cornucopiae* (Rhodophyta, Gigartinaceae) from British Columbia, Canada. J Phycol.

[34] Scrosati R, Servière-Zaragoza E (2000). Ramet dynamics for the clonal seaweed *Pterocladiella capillacea* (Rhodophyta): a comparison with *Chondrus crispus* and with *Mazzaella cornucopiae* (Gigartinales). J Phycol.

[35] Luxoro C, Santelices B (1989). Additional evidence for ecological differences among isomorphic reproductive phases of *Iridaea laminarioides* (Rhodophyta: Girgatinales). J Phycol.

[36] Mach KJ, Tepler SK, Staaf AV, Bohnhoff JC, Denny MW (2011). Failure by fatigue in the field: a model of fatigue breakage for the macroalga *Mazzaella*, with validation. J Exp Biol.

[37] Carrington E, Grace SP, Chopin T (2001). Life history phases and the biomechanical properties of the red alga *Chondrus crispus* (Rhodophyta). J Phycol.

[38] Scrosati R, Mudge B (2004). Effects of elevation, wave exposure, and year on the proportion of gametophytes and tetrasporophytes in *Mazzaella parksii* (Rhodophyta, Gigartinaceae) populations. Hydrobiologia.

[39] Scrosati R, Mudge B (2004). Persistence of gametophyte predominance in *Chondrus crispus* (Rhodophyta, Gigartinaceae) from Nova Scotia after 12 years. Hydrobiologia.

[40] Bellgrove A, Aoki MN (2008). Variation in gametophyte dominance in populations of *Chondrus verrucosus* (Gigartinaceae, Rhodophyta). Phycol Res.

[41] Garbary DJ, Tompkins E, White K, Corey P, Kim J-K (2011). Temporal and spatial variation in the distribution of life history phases of *Chondrus crispus* (Gigartinales, Rhodophyta). Algae.

[42] Dyck LJ, DeWreede RE (2006). Seasonal and spatial patterns of population density in the marine macroalga *Mazzaella splendens* (Gigartinales, Rhodophyta). Phycol Res.

[43] Molina X, Montecino V (1996). Acclimation to UV irradiance in *Gracilaria chilensis* bird, McLachlan & Oliveira (Gigartinales, Rhodophyta). Hydrobiologia.

[44] Gómez I, Figueroa FL, Huovinen P, Ulloa N, Morales V (2005). Photosynthesis of the red alga *Gracilaria chilensis* under natural solar radiation in an estuary in southern Chile. Aquaculture.

[45] Kumar M, Gupta V, Trivedi N, Kumari P, Bijo AJ, Reddy CRK (2011). Desiccation induced oxidative stress and its biochemical responses in intertidal red alga *Gracilaria corticata* (Gracilariales, Rhodophyta). Environ Exp Bot.

[46] Sinha RP, Klisch M, Gröniger A, Häder DP (2000). Mycosporine-like amino acids in the marine red alga *Gracilaria cornea*—effects of UV and heat. Environ Exp Bot.

[47] Jiang H, Gao K, Helbling EW (2008). UV-absorbing compounds in *Porphyra haitanensis* (Rhodophyta) with special reference to effects of desiccation. J Appl Phycol.

[48] Pickering TD, Gordon ME, Tong LJ (1990). Seasonal growth, density, reproductive phenology and agar quality of *Gracilaria sordida* (Gracilariales, Rhodophyta) at Mokomoko Inlet, New Zealand. Hydrobiologia.

[49] Creed JC, Kain JM, Norton TA (1998). An experimental evaluation of density and plant size in two large brown seaweeds. J Phycol.

[50] Steen H, Scrosati R (2004). Intraspecific competition in Fucusserratus and F. evanescens (Phaeophyceae: Fucales) germlings: effects of settlement density, nutrient concentration, and temperature. Mar Biol.

[51] Scrosati R (2005). Review of studies on biomass-density relationships (including self-thinning lines) in seaweeds: Main contributions and persisting misconceptions. Phycol Res.

[52] Lion U, Wiesemeier T, Weinberger F, Beltrán J, Flores V, Faugeron S (2006). Phospholipases and galactolipases trigger oxylipin-mediated wound-activated defence in the red alga *Gracilaria chilensis* against epiphytes. Chem Bio Chem.

[53] Nylund GM, Weinberger F, Rempt M, Pohnert G (2011). Metabolomic assessment of induced and activated chemical defence in the invasive red alga *Gracilaria vermiculophylla*. PLoS One.

[54] Buschmann AH, Santelices B (1987). Micrograzers and spore release in *Iridaea laminarioides* Bory (Rhodophyta: Gigartinales). J Exp Mar Biol Ecol.

[55] Lewis WM (1985). Nutrient scarcity as an evolutionary cause of haploidy. Am Nat.

[56] Mable BK (2001). Ploidy evolution in the yeast *Saccharomyces cerevisiae*: a test of the nutrient limitation hypothesis. J Evol Biol.

[57] Destombe C, Godin J, Nocher M, Richerd S, Valero M (1993). Differences in response between haploid and diploid isomorphic phases of *Gracilaria verrucosa* (Rhodophyta: Gigartinales) exposed to artificial environmental conditions. Hydrobiologia.

[58] Reef R, Pandolfi JM, Lovelock CE (2012). The effect of nutrient enrichment on the growth, nucleic acid concentrations, and elemental stoichiometry of coral reef macroalgae. Ecol Evol.

[59] Camus PA (1992). Size-specific reproductive parameters in red algae: a comparative analysis for two sympatric species from Central Chile. Oecologia.

[60] Hautekèete N-C, Piquot Y, Van Dijk H (2001). Investment in survival and reproduction along a semelparity–iteroparity gradient in the *Beta* species complex. J Evol Biol.

[61] Kain JM, Destombe C (1995). A review of the life history, reproduction and phenology of Gracilaria. J Appl Phycol..

[62] Guillemin ML, Faugeron S, Destombe C, Viard F, Correa JA, Valero M (2008). Genetic variation in wild and cultivated populations of the haploid–diploid red alga Gracilaria chilensis: how farming practices favour asexual reproduction and heterozygosity. Evolution..

